# Help-Seeking Patterns Among the General Population in Singapore: Results from the Singapore Mental Health Study 2016

**DOI:** 10.1007/s10488-020-01092-5

**Published:** 2020-10-15

**Authors:** Saleha Shafie, Mythily Subramaniam, Edimansyah Abdin, Janhavi Ajit Vaingankar, Rajeswari Sambasivam, Yunjue Zhang, Shazana Shahwan, Sherilyn Chang, Anitha Jeyagurunathan, Siow Ann Chong

**Affiliations:** Research Division, Institute of Mental Health, Buangkok Green Medical Park, 10 Buangkok View, Singapore, 539747 Singapore

**Keywords:** Help-seeking, CIDI, Mental health, Service utilisation, Singapore

## Abstract

This study aimed to establish lifetime mental health service utilisation among the general population of Singapore. The sociodemographic correlates of those seeking help from different service provider groups and changes in lifetime mental health service utilisation between 2010 and 2016 among those with mental disorders were also explored. A population-based cross-sectional epidemiological household survey of the Singapore resident population aged 18 years and above was conducted from 2016 to 2018, using the World Mental Health Composite International Diagnostic Interview (CIDI) version 3.0. Data from two cross-sectional population-based studies were used for comparison of lifetime mental health service utilisation in 2010 (n = 6616) and 2016 (n = 6126). Chi square test and multiple logistic regression were used to analyse the data. A total of 6126 respondents completed the study in 2016. Overall 9.3% of the total sample, 32.0% of those with mental disorders, and 5.7% of those not meeting criteria for mental disorders, ever sought help for their mental health issues in their lifetime, from any treatment service sectors. Several sociodemographic characteristics were found to be correlated with different service provider groups. There was no change in mental health service utilisation between 2010 and 2016 for all mental disorders included in this study, with the exception of a significant increase in help sought from professionals in social services, among those with alcohol abuse. Even though the overall help-seeking rates are low, it is encouraging that those seeking help did so from mental health professionals and professionals working in the social services.

**Electronic supplementary material:**

The online version of this article (10.1007/s10488-020-01092-5) contains supplementary material, which is available to authorized users.

## Introduction

Globally more than one billion people were affected by mental or addictive disorders in 2016 (Rehm & Shield 2019). Depressive disorders and anxiety disorders were associated with the highest Disability Adjusted Life Years. In a systematic review on the global prevalence of mental disorders, Steel et al. (2014) found that almost 30% of respondents had experienced a lifetime common mental disorder. Mental disorders are associated with lower quality of life (Coluccia et al. 2016; Dean et al. 2004; Levola et al. 2014), disability (Whiteford et al. 2013), unemployment (Fryers et al. 2005), higher mortality (Walker et al. 2015) and shorter lifespan (Viron & Stern 2010). In 2010, the global cost of mental disorders was estimated to be approximately US$2.5 trillion (Marquez & Saxena 2016), and by 2030, the cost is projected to go up by 240% to US$6.0 trillion (Marquez & Saxena 2016). In the same article, the authors noted that mental disorders were estimated by the World Health Organization (WHO), to account for 30% of non-fatal disease burden worldwide and 10% of overall disease burden, including death and disability (Marquez & Saxena 2016). Given the large numbers of people adversely affected by mental disorders it is thus important to study the utilisation of mental health services among this affected group.

Help-seeking is a behaviour characterized by actively seeking help from other people through obtaining information, advice, treatment, and general support in response to a problem or distressing experience (Rickwood et al. 2005)*.* Although there is an increasing awareness of mental disorders, the use of mental health services by those with mental disorders is still low, especially in Asian countries. The proportion of those with mental disorders who sought treatment was 17.8% in Korea (lifetime) (Park et al. 2017), 26% in Hong Kong (past year) (Lam et al. 2015), and 33.6% in Japan (past 12 months) (Ishikawa et al. 2018). Major barriers to seeking and staying in treatment among those with mental disorders worldwide were found to be low perceived need and attitudinal barriers, where the most common reason was that people wanted to handle the problem on their own (Andrade et al. 2014)*.* Structural barriers such as financial, inconvenience, and difficulties in transportation, were reported for mental disorders which were severe, in high (e.g. New Zealand and USA) and middle-income countries (e.g. Brazil and Mexico), though at a lower proportion compared to attitudinal barriers (Andrade et al. 2014). A systematic review (Gulliver et al. 2010) reported that for young people, the most important barriers were perceived stigma and embarrassment, poor mental health literacy and a preference to rely on themselves rather than seek external help. These findings highlight the importance of examining the patterns of help-seeking in the general population so that mental health services and initiatives can be planned to target the relevant sociodemographic groups that require help but are not seeking it.

Singapore is a multi-ethnic city-state in Southeast Asia with a 3.9 million resident population comprising of Chinese, Malay, Indian and other ethnicities (Department of Statistics Singapore 2018). The Singapore healthcare system consists of the public and private sectors. In the primary healthcare sector, 80% is made up of about 1600 general practitioner (GP) clinics, while the other 20% consists of 20 polyclinics spread across the island (Ministry of Health 2018). Polyclinics provide subsidised primary care, including primary medical treatment, preventive healthcare, and health education. At present, there are seven polyclinics providing mental health services for conditions such as depression and anxiety disorders through multi-disciplinary teams made up of family physicians, nurses, medical social workers, and psychologists. By 2021 about half of the polyclinics in Singapore will provide these subsidised services (The Straits Times 2017a) in line with the Ministry of Health’s (MOH) policy to shift mental health care to the community by making services more readily accessible and to reduce the stigma of getting help (TODAY Online 2017). Besides the primary care sector, there exists a wide range of mental health service providers in Singapore’s private and public sector, including the voluntary welfare organisations (VWOs) (NCSS 2018). These include helplines, counselling services, and support groups that cater to different age groups (i.e. children and youth, adults and seniors) and specific services for different mental health conditions (i.e. addictions, depression and dementia). Public hospitals also provide mental health services along with the Institute of Mental Health (IMH), which is the only tertiary psychiatric hospital in Singapore. In addition to these formal mental health services, people with mental disorders, influenced by cultural and religious beliefs, are also known to seek informal help from traditional and spiritual healers (Chong 2007; Kua 2004) as well as use alternative therapies (Seet et al. 2020).

The first Singapore Mental Health Study was conducted in 2010 (henceforth referred to as SMHS 2010) to establish the prevalence of select mental disorders and their associated sociodemographic correlates (Chong et al. 2012a). The lifetime prevalence of mood, anxiety and alcohol use disorders were 7.0, 3.6, and 3.6%, respectively. Out of those with any mental disorder, 31.7% had sought help from one or more than one service provider for their mental health problems (Chong et al. 2012b).

There is a need to explore changes in the patterns of help-seeking for mental health issues among the general population since the last study in 2010 as programmes have been implemented to raise awareness of mental health and to promote help-seeking over these years. Using data from the second Singapore Mental Health Study (henceforth referred to as SMHS 2016), this research aims to study the (i) pattern of lifetime mental health service utilisation among the general population (includes those with mental disorders and those not meeting criteria for select mental disorders) (ii) sociodemographic correlates of those seeking help at different treatment service provider groups and (iii) change in lifetime mental health service utilisation between 2010 and 2016 among those with mental disorders.

## Method

### Participants and Procedure

SMHS 2016 was conducted following the same methodology and procedures as the SMHS 2010 (Subramaniam et al. 2012). A more detailed methodology of the SMHS 2016 can be found in an earlier paper (Subramaniam et al. 2019). In short, SMHS 2016 is a population-based cross-sectional epidemiological household survey of the Singapore resident population (Singapore Citizens and Permanent Residents) aged 18 years and above who were randomly selected from a national database of residents in Singapore. A disproportionate sampling design was applied where residents belonging to Malay and Indian ethnic groups and those aged 65 and above were over-sampled to ensure a sufficient sample size would be achieved to improve the reliability of estimates for the subgroup analysis. Ethical approval for the study was given by the National Healthcare Group Domain Specific Review Board. A total of 6126 people completed the face-to-face interview conducted by trained interviewers. Interviewers recruited from a survey firm were trained and supervised by the research staff of IMH to conduct face-to-face interviews at respondents’ homes or other places convenient to respondents. Written consent was obtained from respondents, and their legally acceptable representatives (for those under 21 years of age) and in the presence of a witness (for those who are unable to read/write) before starting the survey. Questionnaires were administered using computer-assisted personal interviews (CAPI), which were programmed into tablets. The interviews were conducted from August 2016 to April 2018 in English, Chinese or Malay languages.

### Measures

#### The CIDI 3.0 Diagnostic Section

The Composite International Diagnostic Interview version 3.0 (WHO-CIDI 3.0) (Kessler & Üstün 2004) is a comprehensive, fully-structured interview designed to be used by trained lay interviewers to assess lifetime and 12-month prevalence of mental disorders according to the definitions and criteria of the Diagnostic and Statistical Manual of Mental Disorders, 4th edition (DSM-IV) (APA 2000). The following disorders were assessed in this study using organic exclusion and diagnostic hierarchy rules: generalised anxiety disorder, obsessive compulsive disorder, major depressive disorder, dysthymia, bipolar disorder and alcohol use disorder (alcohol abuse/ dependence). Only the lifetime prevalence of these diagnoses was used in the analyses.

#### The CIDI 3.0 Services Section

Respondents who had ever sought help from a mental health professional or were hospitalized overnight for their mental health problems or had a session of psychological counselling or therapy were asked the following question: ‘Did you ever in your lifetime go to see any of the professionals on this list for problems with your emotions, nerves, mental health or your use of alcohol or drugs?’ Respondents were then shown a list of treatment service providers where they would indicate all that apply to them (i.e. they could choose more than one service provider). The list of treatment service providers contacted were categorised into the following four groups for analysis:

1. Consultation with a mental health professional included a psychiatrist, psychologist, or other mental health professional.
2. Consultation with other medical professionals included general practitioners (GP), other medical doctors, nurses, occupational therapists, or other health professionals.
3. Consultation with a professional from social services, included social worker or counsellor.
4. Consultation with a religious or spiritual advisor/healer.

#### Sociodemographic Variables

Sociodemographic data on age (18–34, 35–49, 50–64 and 65 +), gender (male, female), ethnicity (Chinese, Malay, Indian and Others), marital status (Married, Never married, Divorced/Separated and Widowed), education (Primary and below, Secondary, Pre-U/Junior College, Vocational Institute/ITE, Diploma and University), employment (Employed, Economically inactive and Unemployed) and household income (Below $2000, $2000-$3999, $4000-$5999, $6000–9999 and $10,000 & above) was collected.

#### Statistical Analyses

All estimates were weighted to adjust for over sampling and post-stratified for age and ethnicity distributions between the survey sample and the Singapore resident population in 2014. Weighted mean and standard deviations were calculated for continuous variables and frequencies and percentages for categorical variables. Patterns of service use were examined by computing proportions of treatment, for different settings and according to socio-demographic variables. Data were analysed for those who saw providers in multiple sectors. Univariate analysis using chi-square tests followed by multiple logistic regression analysis were used to study sociodemographic correlates of receiving lifetime treatment in the four service provider groups among those receiving any treatment. Age, gender, ethnicity, marital status, education, employment, and household income were included as independent variables in the regression analysis. Significant changes in the proportion of service use between 2010 and 2016 were tested using chi-square test. Standard errors (SE) and significance tests were estimated using the Taylor series linearization method. Multivariate significance was evaluated using Wald *x*^*2*^ tests based on design corrected coefficient variance–covariance matrices. Statistical significance was evaluated at the 0.05 level using two-sided tests. All statistical analyses were carried out using the statistical analysis system (SAS) software version 9.2 (SAS Institute Inc., NC).

## Results

In all, 6126 respondents completed the study yielding a response rate of 69.5% among the eligible adults. Compared to respondents, non-respondents were more likely to be of Chinese ethnicity and were aged 35 years and above. Males and females were represented equally and the mean (SD) age of the sample was 45.2 (16.4) years. Table 1 shows the socio-demographic distribution of the sample of respondents (n = 6126).

**Table 1:** Demographic distribution of the sample (n = 6126)

Socio-demographic characteristics		n	Unweighted %	Weighted %
Age group (years)	18–34	1707	27.9	30.4
	35–49	1496	24.4	29.6
	50–64	1626	26.5	26.9
	65 +	1297	21.2	13.1
Gender	Women	3058	49.9	50.4
	Men	3068	50.1	49.6
Ethnicity	Chinese	1782	29.1	75.7
	Malay	1990	32.5	12.5
	Indian	1844	30.1	8.7
	Others	510	8.3	3.1
Marital status	Never married	1544	25.2	31.0
	Married	3843	62.7	59.8
	Divorced/Separated	343	5.6	5.2
	Widowed	396	6.5	4.1
Education	Primary and below	1187	19.4	16.3
	Secondary	1648	26.9	23.0
	Pre-U^a^/Junior College	304	5.0	6.0
	Vocational Institute/ITE^b^	508	8.3	6.3
	Diploma	1024	16.7	19.0
	University	1455	23.8	29.4
Employment	Employed	4055	66.2	72.0
	Economically inactive^c^	1716	28.0	22.7
	Unemployed	354	5.8	5.3
Household income (SGD^d^) 1 USD^e^ = 1.39^f^ SGD	Below 2000	1147	21.0	16.5
	2000–3999	1331	24.4	20.0
	4000–5999	1113	20.4	21.4
	6000–9999	1003	18.4	21.8
	10, 000 & above	861	15.8	20.3

^a^Pre-U: Pre-University

^b^ITE: Institute of Technical Education

^c^includes retirees, homemakers and students

^d^SGD: Singapore Dollars

^e^USD: US Dollar

^f^Currency exchange rate accessed at xe.com on 15/6/2020

### Pattern of Lifetime Mental Health Service Utilisation among the General Population

The treatment service sectors that the respondents sought help for their mental health issues at least once in their lifetime (including those who sought help from multiple service providers) is shown in Table 2. Overall among the Singapore general population, 9.3% of respondents had ever sought help from any of the four treatment service sectors in their lifetime. Among respondents who met criteria for at least one mood, anxiety or alcohol use disorder in their lifetime, about one in three (32.0%) ever sought help from any of the four treatment service provider groups in their lifetime. Among those with anxiety and mood disorders (except dysthymia), the highest proportion of help sought in their lifetime was from mental health professionals. Among those with dysthymia and alcohol abuse, the highest proportion of help sought in their lifetime was from social services professionals. And lastly among those with alcohol dependence, religious or spiritual advisor/healer were the main source of help-seeking. Among those who did not meet the criteria for any of the mood, anxiety, and alcohol disorders, 5.7% of them had ever sought help from any of the four treatment service provider groups in their lifetime.

**Table 2:** Pattern of lifetime service use among the general population

Type of Disorder	Lifetime weighted Prevalence	Any service provider	Any mental health professional	Any other medical health professional	Any professional in social services	Any religious or spiritual advisor/ healer
	n (%^a^)	n (%^a^)	n (%^a^)	n (%^a^)	n (%^a^)	n (%^a^)
Mood disorders						
MDD	346 (6.3)	135 (39.7)	**63 (19.9)**	34 (9.6)	61 (18.2)	36 (11.0)
Dysthymia	27 (0.3)	16 (54.6)	6 (21.4)	4 (16.0)	**11 (24.3)**	3 (17.9)
Bipolar	105 (1.6)	55 (49.0)	**32 (37.7)**	17 (16.5)	25 (14.7)	12 (9.3)
Anxiety disorders						
GAD	101 (1.6)	54 (55.7)	**29 (33.7)**	17 (11.1)	21 (14.8)	13 (10.0)
OCD	217 (3.6)	62 (30.3)	**30 (20.2)**	18 (7.5)	29 (12.0)	17 (5.1)
Alcohol use disorders						
Alcohol abuse	247 (4.1)	58 (24.6)	32 (13.9)	19 (6.9)	**30 (15.1)**	10 (5.8)
Alcohol dependence	42 (0.5)	15 (33.7)	5 (12.3)	3 (3.4)	6 (7.7)	**5 (15.0)**
Composite						
Any of the above disorders	846 (13.9)	271 (32.0)	**132 (17.0)**	77 (8.3)	123 (13.5)	63 (7.5)
None of the above disorders	5280 (86.1)	315 (5.7)	138 (2.5)	83 (1.3)	**139 (2.7)**	46 (0.7)
Total sample	6126 (100)	586 (9.3)	**270 (4.5)**	160 (2.3)	262 (4.2)	109 (1.6)

Numbers in bold are the highest proportion of service group utilised for each disorder

^a^Weighted %

### Sociodemographic Correlates Associated with the Different Treatment Service Sectors

Table 3 shows the sociodemographic correlates associated with those seeking help at the four different treatment service sectors for their mental health problems using multiple logistic regression analysis.

**Table 3:** Sociodemographic correlates of lifetime help-seeking in the four treatment service sectors among the general population (n = 6126)

Variable	Any service provider OR^a^ (p value)	Any mental health professional OR^a^ (p value)	Any other medical health professional OR^a^ (p value)	Any professional in social services OR^a^ (p value)	Any religious or spiritual advisor/healer OR^a^ (p value)
Age group					
18–34	Ref	Ref	Ref	Ref	Ref
35–49	0.8 (0.240)	1.3 (0.399)	1.1 (0.824)	**0.4 (0.001)**	1.2 (0.676)
50–64	0.6 (0.074)	0.8 (0.563)	1.4 (0.491)	**0.3 (0.000)**	0.6 (0.462)
65 +	**0.4 (0.003)**	0.6 (0.264)	0.7 (0.596)	**0.02 (0.000)**	0.7 (0.707)
Gender					
Female	Ref	Ref	Ref	Ref	Ref
Male	0.8 (0.055)	1.1 (0.727)	0.8 (0.298)	**0.6 (0.029)**	1.7 (0.072)
Ethnicity					
Chinese	Ref	Ref	Ref	Ref	Ref
Malay	1.1 (0.572)	0.9 (0.516)	1.3 (0.334)	1.0 (0.88)	1.5 (0.202)
Indian	1.2 (0.161)	0.9 (0.785)	1.2 (0.491)	1.2 (0.473)	1.3 (0.336)
Others	**1.8 (0.002)**	**2.0 (0.002)**	**2.2 (0.011)**	1.6 (0.074)	**2.3 (0.030)**
Marital status					
Married	Ref	Ref	Ref	Ref	Ref
Never married	1.3 (0.129)	1.6 (0.092)	1.8 (0.174)	1.2 (0.358)	1.4 (0.446)
Divorced/Separated	**2.3 (0.001)**	**2.2 (0.028)**	2.2 (0.084)	**2.0 (0.049)**	**3.8 (0.003)**
Widowed	0.6 (0.129)	0.6 (0.314)	1.3 (0.619)	**0.3 (0.042)**	0.9 (0.847)
Education					
University	Ref	Ref	Ref	Ref	Ref
Primary and below	0.9 (0.780)	1.3 (0.575)	0.7 (0.523)	1.1 (0.812)	0.4 (0.161)
Secondary	1.0 (0.844)	0.9 (0.84)	0.8 (0.629)	1.2 (0.623)	0.7 (0.600)
Pre-U/Junior College	1.0 (0.885)	0.7 (0.573)	1.1 (0.858)	1.5 (0.293)	0.9 (0.918)
Vocational Institute/ITE	1.1 (0.842)	0.9 (0.811)	1.2 (0.77)	1.6 (0.243)	0.6 (0.538)
Diploma	1.2 (0.380)	1.0 (0.866)	0.9 (0.851)	1.0 (0.995)	1.2 (0.594)
Employment					
Employed	Ref	Ref	Ref	Ref	Ref
Economically inactive	1.0 (0.777)	0.9 (0.697)	1.0 (0.895)	0.9 (0.784)	1.0 (0.942)
Unemployed	**2.0 (0.007)**	**3.4 (0.000)**	1.2 (0.676)	1.2 (0.677)	2.5 (0.061)
Household income (SGD)					
Below $2000	Ref	Ref	Ref	Ref	Ref
$2000-$3999	0.8 (0.292)	**0.5 (0.044)**	1.4 (0.352)	0.7 (0.205)	1.2 (0.663)
$4000-$5999	0.8 (0.438)	0.9 (0.803)	1.2 (0.583)	0.5 (0.064)	0.8 (0.697)
$6000–9999	0.9 (0.623)	0.7 (0.292)	1.0 (0.943)	0.7 (0.315)	1.5 (0.381)
$10,000 & above	0.8 (0.425)	0.8 (0.532)	0.9 (0.853)	0.6 (0.114)	1.2 (0.825)

Bolded numbers are statistically significant where p value < 0.05

^a^Adjusted OR was derived using multiple logistic regression analysis after controlling for age, gender, ethnicity, marital status, education, employment and household income

The older age groups [35–49 years (OR: 0.4), 50–64 years (OR: 0.3) and 65 years and above (OR: 0.02)] were significantly associated with lower odds of seeking help from professionals in social services compared to those aged 18–34 years. Those aged 65 years and above were also significantly associated with lower odds of seeking help from any service providers (OR: 0.4) compared to those aged 18–34 years. Males were significantly associated with lower odds of seeking help from professionals from social services (OR: 0.6) compared to females.

Those who were divorced/separated were significantly associated with higher odds of seeking help from any service provider (OR: 2.3), mental health professionals (OR: 2.2), professionals from social services (OR: 2.0), and religious or spiritual advisor/healer (OR: 3.8) compared to those who were married. Those who were widowed were significantly associated with lower odds of seeking help from professionals from social services (OR: 0.3) compared to those who were married. Those who were unemployed were significantly associated with higher odds of seeking help from any service provider (OR: 2.0) and mental health professionals (OR: 3.4) compared to those who were employed.

### Changes in Pattern of Lifetime Service Use from 2010 to 2016 in the Four Treatment Service Provider Groups

There were no significant changes observed in most mental disorders across the different service providers groups except for alcohol abuse and alcohol dependence (Table 4). Among those diagnosed with alcohol abuse, the proportion of people who sought help from social services increased significantly from 7.2% in 2010 to 15.2% in 2016 (p value = 0.049). Among those diagnosed with alcohol dependence, the proportion of people who sought help from social services decreased significantly from 28.0% in 2010 to 7.8% in 2016 (p value = 0.018).

**Table 4:** Change in pattern of lifetime service use from 2010 (Total n = 874) to 2016 (Total n = 846) in the four service provider groups by lifetime mental disorder diagnosis

Diagnoses	Any mental health professional seen					Any other medical health professional seen					Any professional in social services					Any religious or spiritual advisor/healer				
	2010		2016			2010		2016			2010		2016			2010		2016		
	n	%	n	%	P value	N	%	n	%	P value	n	%	n	%	P value	n	%	n	%	P value
Mood	88	21.7	97	23.6	0.644	67	11.4	54	11.4	0.988	81	16.4	86	17.2	0.840	46	9.3	49	10.7	0.629
MDD	65	18.1	63	19.9	0.666	55	11.5	34	9.6	0.553	62	14.9	61	18.1	0.412	33	7.9	36	11.1	0.301
Bipolar	23	38.3	32	37.8	0.960	12	10.4	17	16.8	0.367	19	23.4	25	14.7	0.248	13	15.7	12	9.3	0.340
Anxiety	42	18.0	53	22.9	0.342	24	10.1	32	9.0	0.754	34	12.7	47	12.5	0.963	23	10.9	27	6.7	0.199
GAD	17	31.0	29	34.0	0.784	12	20.8	17	11.3	0.234	15	26.0	21	14.6	0.193	12	20.5	13	10.0	0.188
OCD	29	14.6	30	20.3	0.292	15	8.7	18	7.6	0.757	21	9.2	29	11.9	0.503	13	7.6	17	5.1	0.429
Alcohol use disorder	25	9.1	37	13.8	0.221	15	5.3	22	6.6	0.635	30	9.8	36	14.3	0.254	12	4.5	15	6.9	0.413
Alcohol abuse	18	7.0	32	14.1	0.076	14	5.8	19	7.0	0.701	18	7.2	30	15.2	**0.049**	9	4.5	10	5.8	0.671
Alcohol dependence	7	23.2	5	12.2	0.386	1	1.7	3	3.5	0.531	12	28.0	6	7.8	**0.018**	3	4.4	5	15.2	0.136
Any disorder	117	15.7	132	17.1	0.608	86	9.1	77	8.3	0.705	111	12.7	123	13.5	0.755	59	7.6	63	7.6	0.980

Bolded numbers are statistically significant where p value < 0.05

## Discussion

Slightly less than one-third (32.0%) of those who had ever been diagnosed with a mental disorder had sought help from any of the service providers for their condition, similar to the finding in SMHS 2010 where 31.7% had ever sought help (Chong et al. 2012b). This slight but insignificant change in help-seeking prevalence may indicate that overall, there has not been an increase in help-seeking even though there was a significant increase in the prevalence of mental disorders in the general population from 2010 to 2016 (Subramaniam et al., 2019).

Respondents who experienced mental health problems but did not meet criteria for any mood, anxiety or alcohol use disorder, sought help at rates that were expectedly much lower than those who met criteria for mental disorders. Among those with any mood, anxiety, or alcohol use disorders, the highest proportion of respondents sought help from mental health professionals followed by professionals from social services, other medical health professionals, and lastly, religious or spiritual advisors/healers.

Medical health professionals especially in the primary care setting, are important sources of help-seeking for people with mental illnesses. A study on pathways to care of psychiatric outpatients in IMH (Jeyagurunathan et al., 2018) found that the first point of contact for their mental health issues were primary care clinicians (e.g. GP, polyclinic doctors). In Singapore, about 190 GP partners have been trained to diagnose and support persons with mental health conditions in the community, supported by allied health-led community intervention teams (Ministry of Health 2019). This effort along with the plan to increase the number of mental health clinics in polyclinics by 2021, should further encourage early help-seeking among the general population.

Singapore’s population includes people from different cultural and religious backgrounds, where each community would have their own cultural/religious beliefs regarding mental health. The different ethnic groups in Singapore were found to vary in their perception of mental disorders (Subramaniam et al. 2018). For some who view their mental or emotional problems to be related to spirituality, religious participation or other coping methods are sought instead of formal mental health care (Picco et al. 2013). Picco et al. (2013) found that religious and spiritual advisors were an important source of help among those with mental disorders and that the latter were satisfied with the support received. Therefore efforts need to be made to approach each community accordingly. For example, “socio-emotional needs including relationship counselling and spiritual upliftment” was found to be one of the main areas that the Malay/Muslim community in Singapore sought religious teachers for advice (Majlis Ugama Islam Singapura (MUIS), 2020). It is thus important to engage these religious teachers by providing information on mental health so that they would know how to respond to and be able to refer members of the community with mental health issues to the relevant services and mental health professionals.

Overall among the general population, those in the older age groups were less likely to be associated with seeking help for their mental health issues from professionals in the social services sector compared to those aged 18–34 years. In other words those in the youngest age group were more likely to seek help from counsellors and social workers when they encounter mental health issues. Young people in Singapore aged 18–34 were found to be significantly associated with openness to seeking professional help in a study on mental health literacy (Picco et al. 2016b). Older adults reportedly do not use mental health services due to a lack of perceived need and self-sufficiency beliefs where they prefer to handle their problems on their own (Mackenzie et al. 2010). Besides increasing mental health literacy in the community, efforts should also be made to reduce stigma related to seeking help as stigma has been found to have a small- to moderate-sized negative effect on help-seeking (Clement et al. 2015).

Studies have found that those who were divorced/separated were more likely to seek help for their mental health issues (Andrews et al. 2001; Bracke et al. 2010; Lam et al. 2015). In this study those who were currently divorced/separated were more likely to seek help in their lifetime from any service providers, mental health professionals, professionals from the social services and religious or spiritual advisors/healers. This can be attributed to the psychological distress from the relationship breakdown (Bracke et al. 2010; Parslow & Jorm 2000), which results in those divorced/separated seeking help due to social or emotional problems (Colman et al. 2012).

Bijl & Ravelli (2000) also found that unemployed people were more likely to use mental healthcare. Those who were employed may have less daytime available to spare (Wallerblad et al. 2012) to utilise mental health services available and less attention has been given to their mental health needs (Khlat et al. 2013; Lam et al. 2015). It might also be due to stigma and the fear of being dismissed from their jobs if their employers find out that they have mental disorders (Honkonen et al. 2007), which may have led to low help-seeking in this group. Those who were employed tended to seek informal help, while the unemployed were more likely to seek formal and informal help (Brown et al. 2014). The unemployed may have more time (Honkonen et al. 2007), have less social support, and lack financial means (Khlat et al. 2013). These may seem like barriers but on the contrary, it facilitates help-seeking as they might be encouraged to report mental health issues they face during inevitable visits to the doctor, thus facilitating help-seeking for their mental health problems.

The significant increase in the proportion of those with alcohol abuse seeking help from the social services sector from 2010 to 2016 may be due to the high recognition of the disorder in the community. Alcohol abuse was one of the three most recognised mental disorders among the Singapore general population (Chong et al. 2016), but it was also associated with the highest stigma and viewed as a weakness and not a real illness (Subramaniam et al. 2017). The top three sources of help recommended for alcohol abuse were family and friends, doctor or general practitioner, and counsellor or have counselling (Picco et al. 2016a). On the other hand, there was a significant decrease in the proportion of those with alcohol dependence seeking help from the social services sector. However, due to the small numbers of people seeking help in the two studies, we are unable to explain this finding further.

There was no significant change observed in terms of the other mental disorders and treatment service sectors between the two surveys in 2010 and 2016. This might reflect a stable proportion of the population seeking help over their lifetime. As part of the 5-year Community Mental Health Masterplan proposed by the Ministry of Health (MOH) (The Straits Times 2017b), frontline staff of government agencies, community partners and social services agencies, whose course of work largely involve interacting with members of the public, will be trained to identify and respond to persons with mental health issues. This should improve early identification of mental health symptoms, creating a continuous outreach effort in turn increasing help-seeking in the community.

### Limitations

Several limitations in this study should be mentioned. There was a non-response rate of about 30%, which could lead to an underestimation of the prevalence rate as individuals who refused participation may be unwilling to report that they suffer from mental disorders due to stigma. In addition, participants may also under-report or deny the symptoms they have experienced due to recall and social desirability biases, which are inherent in an interviewer-administered surveys. This study only looked at select mental disorders. Therefore respondents who were classified as having no disorder could have met criteria for other disorders that were not assessed in this study. Lifetime service use data was used in this study to attempt to detect change in pattern of service use over a 6-year period. Last 12-month service use data would have been more suitable to compare changes in service use over the two time periods, however both last 12-month prevalence and help-seeking rates were low and did not lend themselves well to analysis. The authors also acknowledge that those not meeting 12-month criteria of a mental disorder may still be undergoing maintenance treatment as recommended by their treatment provider (Pierre et al. 2007). Lastly, there might be Type 1 error inflation due to multiple comparisons. However the authors did not adjust this as it is inconclusive whether the significance level from multiple comparison should be adjusted (O’Keefe 2003).

In spite of these limitations strengths of the study include the use of the same methodology and structured questionnaire which allowed for observation of changes between the two surveys. A representative sample of the general population was obtained through random sampling; the large sample size and availability of the survey in the main local languages makes our results generalizable to Singapore’s population. High quality of data collection was ensured through rigorous training and evaluation of interviewers, and conducting regular checks on the processes and field observations over the course of the study.

## Conclusion

One in three people with mental disorders ever sought help in their lifetime for their mental health issues, similar to the rate found in 2010. Though the rates are low, it is encouraging to note that the general population (those with mental disorders and those not meeting criteria for select mental disorders) ever sought help in their lifetime from mental health professionals and professionals from the social services. More attention should be given to those who are employed and the older age groups in mental health initiatives to encourage these groups to seek help when they encounter mental health issues. Compared to findings in SMHS 2010 there was significant increase among those with alcohol abuse seeking help from social services professionals. Current initiatives like the five-year Community Mental Health Masterplan led by the Ministry of Health and other programmes by other mental health organisations in Singapore should be continued and supported to encourage help-seeking amongst people with mental health issues.

## Electronic supplementary material

Below is the link to the electronic supplementary material.

Supplementary file1 (DOCX 16 kb)

## References

[CR100] American Psychiatric Association. (2000). *Diagnostic and statistical manual of mental disorders, fourth edition, text revision (DSM-IV-TR*^*®*^*)*. Washington, DC: Author.

[CR1] Andrade LH, Alonso J, Mneimneh Z, Wells JE, Alhamzawi A, Borges G, PosadaVilla J (2014). Barriers to mental health treatment: results from the WHO World Mental Health surveys. Psychological Medicine.

[CR2] Andrews G, Issakidis C, Carter G (2001). Shortfall in mental health service utilisation. British Journal of Psychiatry.

[CR3] Bijl, R. V, & Ravelli, A. (2000). Psychiatric morbidity, service use, and need for care in the general population: Results of the Netherlands Mental Health Survey and Incidence study. *American Journal of Public Health*, *90*(4), 602–607. Retrieved November 12, 2018, from https://www.ncbi.nlm.nih.gov/pmc/articles/PMC1446190/pdf/10754976.pdf 10.2105/ajph.90.4.602 PMC1446190 10754976

[CR4] Bracke PF, Colman E, Symoens SA, Praag LV (2010). Divorce, divorce rates, and professional care seeking for mental health problems in Europe: A cross-sectional population-based study. BMC Public Health.

[CR5] Brown JS, Evans-Lacko S, Aschan L, Henderson MJ, Hatch SL, Hotopf M (2014). Seeking informal and formal help for mental health problems in the community: A secondary analysis from a psychiatric morbidity survey in South London. BMC Psychiatry.

[CR6] Chong SA (2007). Mental health in Singapore: A quiet revolution?. Annals of the Academy of Medicine Singapore.

[CR7] Chong SA, Abdin E, Picco L, Pang S, Jeyagurunathan A, Vaingankar JA, Subramaniam M (2016). Recognition of mental disorders among a multiracial population in Southeast Asia. BMC Psychiatry.

[CR8] Chong, S. A., Abdin, E., Vaingankar, J. A., Heng, D., Sherbourne, C., Yap, M., Subramaniam, M. (2012a). A Population-based survey of mental disorders in Singapore. *Annals Academy of Medicine*, *41*(2), 49–66. Retrieved October 16, 2018, from https://open-access.imh.com.sg/bitstream/123456789/4547/1/A Population-based Survey of Mental Disorders in Singapore.pdf 22498852

[CR9] Chong SA, Abdin E, Vaingankar JA, Kwok KW, Subramaniam M (2012). Where do people with mental disorders in Singapore go to for help?. Annals of the Academy of Medicine Singapore.

[CR10] Clement S, Schauman O, Graham T, Maggioni F, Evans-Lacko S, Bezborodovs N, Thornicroft G (2015). What is the impact of mental health-related stigma on help-seeking? A systematic review of quantitative and qualitative studies. Psychological Medicine.

[CR11] Colman, E., Symoens, S., & Bracke, P. (2012). Professional health care use and subjective unmet need for social or emotional problems: a cross-sectional survey of the married and divorced population of Flanders. *Health Services Research*, *12*, 420. Retrieved February 21, 2019, from https://www.biomedcentral.com/1472-6963/12/420. 10.1186/1472-6963-12-420 PMC3562142 23173927

[CR12] Coluccia A, Fagiolini A, Ferretti F, Pozza A, Costoloni G, Bolognesi S, Goracci A (2016). Adult obsessive–compulsive disorder and quality of life outcomes: A systematic review and meta-analysis. Asian Journal of Psychiatry.

[CR13] Dean BB, Gerner D, Gerner RH (2004). A systematic review evaluating health-related quality of life, work impairment, and healthcare costs and utilization in bipolar disorder. Current Medical Research and Opinion.

[CR14] Department of Statistics Singapore. (2018). *Population Trends 2018*. Singapore. Retrieved December 5, 2018, from https://www.singstat.gov.sg/tablebuilder.

[CR15] Fryers T, Melzer D, Jenkins R, Brugha T (2005). The distribution of the common mental disorders: Social inequalities in Europe. Clinical Practice and Epidemiology in Mental Health.

[CR16] Gulliver A, Griffiths KM, Christensen H (2010). Perceived barriers and facilitators to mental health help-seeking in young people: a systematic review. BMC Psychiatry.

[CR17] Honkonen T, Virtanen M, Ahola K, Kivimäki M, Pirkola S, Isometsä E, Lönnqvist J (2007). Employment status, mental disorders and service use in the working age population. Scandinavian Journal of Work, Environment and Health.

[CR18] Ishikawa H, Tachimori H, Takeshima T, Umeda M, Miyamoto K, Shimoda H, Kawakami N (2018). Prevalence, treatment, and the correlates of common mental disorders in the mid 2010′s in Japan: The results of the world mental health Japan 2nd survey. Journal of Affective Disorders.

[CR19] Jeyagurunathan A, Abdin E, Shafie S, Wang P, Chang S, Ong HL, Subramaniam M (2018). Pathways to care among psychiatric outpatients in a tertiary mental health institution in Singapore. International Journal of Social Psychiatry.

[CR20] Kessler, R. C., & Üstün, T. B. (2004). The World Mental Health (WMH) Survey Initiative Version of the World Health Organization (WHO) Composite International Diagnostic Interview (CIDI). *International Journal of Methods in Psychiatric Research, 13*(2), 93-121. Retrieved December 1, 2018, from https://www.hcp.med.harvard.edu/wmh/publishedpaper_kessler_wmhcidi.pdf 10.1002/mpr.168 PMC6878592 15297906

[CR21] Khlat M, Legleye S, Sermet C (2013). Factors influencing report of common mental health problems among psychologically distressed adults. Community Mental Health Journal.

[CR22] Kua E-H (2004). Focus on psychiatry in Singapore. British Journal of Psychiatry.

[CR23] Lam LC-W, Wong CS-M, Wang M-J, Chan W, Chen EY-H, Ng RM-K, Bebbington P (2015). Prevalence, psychosocial correlates and service utilization of depressive and anxiety disorders in Hong Kong: the Hong Kong Mental Morbidity Survey (HKMMS). Social Psychiatry and Psychiatric Epidemiology.

[CR24] Levola J, Aalto M, Holopainen A, Cieza A, Pitkänen T (2014). Health-related quality of life in alcohol dependence: A systematic literature review with a specific focus on the role of depression and other psychopathology. Nordic Journal of Psychiatry.

[CR25] Mackenzie CS, Pagura J, Sareen J (2010). Correlates of perceived need for and use of mental health services by older adults in the collaborative psychiatric epidemiology surveys. The American Journal of Geriatric Psychiatry.

[CR26] Majlis Ugama Islam Singapura (MUIS). (2020). *Strengthening Religious Leadership for a Community of Success*. Retrieved September 14, 2020, from https://www.muis.gov.sg/-/media/Files/Corporate-Site/COFA/COFA-Report.pdf?la=en&hash=7FE404772359FE0731A82A3424A1BDC558943F33.

[CR27] Marquez, P. V, & Saxena, S. (2016). Making Mental Health a Global Priority. *Cerebrum: The Dana Forum on Brain Science*, *2016*, cer 10–1. Retrieved December 14, 2019, from https://www.ncbi.nlm.nih.gov/pubmed/28058091 PMC5198754 28058091

[CR28] Ministry of Health. (2018). Primary Healthcare Services. Retrieved August 30, 2018, from https://www.moh.gov.sg/our-healthcare-system/healthcare-services-and-facilities/primary-healthcare-services

[CR29] Ministry of Health. (2019). SPEECH BY DR AMY KHOR, SENIOR MINISTER OF STATE FOR HEALTH, AT THE MINISTRY OF HEALTH COMMITTEE OF SUPPLY DEBATE 2019, ON WEDNESDAY 6 MARCH 2019. Retrieved May 21, 2019, from https://www.moh.gov.sg/news-highlights/details/speech-by-dr-amy-khor-senior-minister-of-state-for-health-at-the-ministry-of-health-committee-of-supply-debate-2019-on-wednesday-6-march-2019

[CR30] NCSS. (2018). *Resource Directory on Mental Health Services in Singapore*. Retrieved October 23, 2018 from https://www.ncss.gov.sg/NCSS/media/NCSS-Documents-and-Forms/NCSS Internal Documents/Directory-on-Mental-Health-Services.pdf.

[CR31] O’Keefe DJ (2003). Colloquy: Should familywise alpha be adjusted? Against familywise alpha adjustment. Human Communications Research.

[CR32] TODAY Online. (2017). More coordinated stream of care, services for those with mental health issues. Retrieved August 30, 2018, from https://www.gov.sg/news/content/today-online---more-coordinated-stream-of-care-services-for-those-with-mental-health-issues.

[CR33] Park S, Lee Y, Jeong Seong S, Man Chang S, Young Lee J, Jin Hahm B, Pyo Hong J (2017). A cross-sectional study about associations between personality characteristics and mental health service utilization in a Korean national community sample of adults with psychiatric disorders. BMC Psychiatry.

[CR34] Parslow, R. A., & Jorm, A. F. (2000). Who uses mental health services in Australia? An analysis of data from the National Survey of Mental Health and Wellbeing. *Australian and New Zealand Journal of Psychiatry*, *34*, 997–1008. Retrieved November 12, 2018 from https://citeseerx.ist.psu.edu/viewdoc/download?doi=10.1.1.899.9840&rep=rep1&type=pdf. 10.1080/000486700276 11127632

[CR35] Picco L, Abdin E, Chong A, Pang S, Vaingankar JA, Sagayadevan V, Subramaniam M (2016). Beliefs about help seeking for mental disorders: Findings from a mental health literacy study in Singapore. Psychiatric Services.

[CR36] Picco L, Abdin E, Chong SA, Pang S, Shafie S, Chua BY, Subramaniam M (2016). Attitudes toward seeking professional psychological help: Factor structure and socio-demographic predictors. Front. Psychol.

[CR37] Picco L, Subramaniam M, Abdin E, Vaingankar JA, Zhang Y, Chong SA (2013). Roles of religious and spiritual advisors among adults in Singapore with mental illnesses. Psychiatric Services.

[CR38] Pierre, B., Keller, M. B., Pollack, M. H., Thase, M. E., Zajecka, J. M., & Dunner, D. L. (2007). Preventing recurrent depression: long-term treatment for major depressive disorder. *The Journal of Clinical Psychiatry*, *68*(3), e06. Retrieved September 13, 2020 from https://pubmed.ncbi.nlm.nih.gov/17388700/. 17388700

[CR39] Rehm J, Shield KD (2019). Global burden of disease and the impact of mental and addictive disorders. Current Psychiatry Reports.

[CR40] Rickwood, D., Deane, F. P., Wilson, C. J., & Ciarrochi, J. (2005). Young people’s help-seeking for mental health problems. *Australian E-Journal for the Advancement of Mental Health*, *4*(3). Retrieved October 30, 2018 from www.auseinet.com/journal/vol4iss3suppl/rickwood.pdfwww.auseinet.com/journal

[CR41] Seet V, Abdin E, Vaingankar JA, Shahwan S, Chang S, Lee B, Subramaniam M (2020). The use of complementary and alternative medicine in a multi-ethnic Asian population: Results from the 2016 Singapore Mental Health Study. BMC Complementary Medicine and Therapies.

[CR42] Steel Z, Marnane C, Iranpour C, Chey T, Jackson JW, Patel V, Silove D (2014). The global prevalence of common mental disorders: A systematic review and meta-analysis 1980–2013. International Journal of Epidemiology.

[CR43] Subramaniam M, Abdin E, Jeyagurunathan A, Chang S, Samari E, Shafie S, Chong SA (2018). Exploration of illness perception among patients with mental illness in a multi-ethnic Asian sample. Psychiatry Research.

[CR44] Subramaniam M, Abdin E, Picco L, Pang S, Shafie S, Vaingankar JA, Chong SA (2017). Stigma towards people with mental disorders and its components-a perspective from multi-ethnic Singapore. Epidemiology and Psychiatric Sciences.

[CR45] Subramaniam M, Abdin E, Vaingankar JA, Shafie S, Chua BY, Sambasivam R, Chong SA (2019). Tracking the mental health of a nation: prevalence and correlates of mental disorders in the second Singapore mental health study. Epidemiology and Psychiatric Sciences.

[CR46] Subramaniam M, Vaingankar J, Heng D, Kwok KW, Lim YW, Yap M, Chong SA (2012). The Singapore Mental Health Study: an overview of the methodology. International Journal of Methods in Psychiatric Research.

[CR47] The Straits Times. (2017a). Mental health cases treated at polyclinics. Retrieved August 30, 2018, from https://www.straitstimes.com/singapore/health/mental-health-cases-treated-at-polyclinics

[CR48] The Straits Times. (2017b). New moves to enhance community care for mental health. Retrieved September 3, 2018, from https://www.straitstimes.com/singapore/new-moves-to-enhance-community-care-for-mental-health

[CR49] Viron MJ, Stern TA (2010). The impact of serious mental illness on health and healthcare. Psychosomatics.

[CR50] Walker ER, McGee RE, Druss BG (2015). Mortality in mental disorders and global disease burden implications: A systematic review and meta-analysis. JAMA Psychiatry.

[CR51] Wallerblad A, Möller J, Forsell Y (2012). Care-seeking pattern among persons with depression and anxiety: A population-based study in Sweden. International Journal of Family Medicine 2012.

[CR52] Whiteford HA, Degenhardt L, Rehm J, Baxter AJ, Ferrari AJ, Erskine HE, Vos T (2013). Global burden of disease attributable to mental and substance use disorders: findings from the Global Burden of Disease Study 2010. The Lancet.

