# Supplementary material for: Help-Seeking Patterns Among the General Population in Singapore: Results from the Singapore Mental Health Study 2016
Source: Adm Policy Ment Health. 2020 Oct 15;48(4):586–96. doi: 10.1007/s10488-020-01092-5 (PMC8192323; doi:10.1007/s10488-020-01092-5)
Supplement: Supplementary file 1 — Supplementary file1 (DOCX 16 kb) [file 10488_2020_1092_MOESM1_ESM.docx]

Supplementary Table. Sociodemographic correlates of lifetime help-seeking in the four treatment service sectors among those with any CIDI diagnosis (n=846)

| **Variable** | **Any service provider OR^a^ (p value)** | **Any mental health professional  OR^a^ (p value)** | **Any other medical health professional OR^a^ (p value)** | **Any professional in a social services setting OR^a^ (p value)** | **Any religious or spiritual advisor/healer OR^a^ (p value)** |
| --- | --- | --- | --- | --- | --- |
|  |  |  |  |  |  |
| Age group |  |  |  |  |  |
| 18-34 | Ref | Ref | Ref | Ref | Ref |
| 35-49 | 1.4 (0.232) | **2.8 (0.014)** | 2.8 (0.076) | 0.8 (0.631) | **3.2 (0.048)** |
| 50-64 | 1.2 (0.583) | 1.7 (0.266) | **3.9 (0.027)** | 0.5 (0.167) | 1.4 (0.659) |
| 65+ | 1.0 (0.959) | 1.5 (0.584) | 2.3 (0.329) | 0.3 (0.213) | 3.2 (0.204) |
|  |  |  |  |  |  |
| Gender |  |  |  |  |  |
| Female | Ref | Ref | Ref | Ref | Ref |
| Male | **0.5 (0.005)** | 0.7 (0.188) | 0.6 (0.164) | 0.7 (0.319) | 0.9 (0.840) |
|  |  |  |  |  |  |
| Ethnicity |  |  |  |  |  |
| Chinese | Ref | Ref | Ref | Ref | Ref |
| Malay | 0.9 (0.664) | 0.6 (0.083) | 1.1 (0.737) | 1.3 (0.382) | 1.0 (0.910) |
| Indian | 1.0 (0.931) | 0.7 (0.120) | 1.0 (0.997) | 1.1 (0.676) | 1.1 (0.812) |
| Others | 1.7 (0.090) | 1.9 (0.068) | 1.9 (0.163) | **2.4 (0.022)** | 1.7 (0.270) |
|  |  |  |  |  |  |
| Marital status |  |  |  |  |  |
| Married | Ref | Ref | Ref | Ref | Ref |
| Never married | 1.3 (0.404) | 1.9 (0.106) | 1.7 (0.363) | 1.4 (0.286) | 2.4 (0.133) |
| Divorced/Separated | 2.1 (0.070) | 1.8 (0.222) | 1.5 (0.483) | 1.5 (0.371) | 2.0 (0.237) |
| Widowed | **0.2 (0.017)** | 0.4 (0.256) | 0.2 (0.123) | 0.1 (0.065) | 0.3 (0.339) |
|  |  |  |  |  |  |
| Employment |  |  |  |  |  |
| Employed | Ref | Ref | Ref | Ref | Ref |
| Economically inactive | 1.3 (0.503) | 1.1 (0.889) | 0.9 (0.928) | 2.0 (0.075) | 1.3 (0.662) |
| Unemployed | 1.9 (0.100) | **4.0 (0.001)** | 0.8 (0.626) | 1.2 (0.771) | 1.2 (0.737) |
